# Left atrial strain and clinical outcome in patients with significant mitral regurgitation after surgical mitral valve repair

**DOI:** 10.3389/fcvm.2022.985122

**Published:** 2022-10-04

**Authors:** Se-Eun Kim, Dae-Young Kim, Jiwon Seo, Iksung Cho, Geu-Ru Hong, Jong-Won Ha, Chi Young Shim

**Affiliations:** ^1^Division of Cardiology, Severance Cardiovascular Hospital, Yonsei University College of Medicine, Seoul, South Korea; ^2^Department of Cardiology, CHA Bundang Medical Center, CHA University, Seongnam, South Korea

**Keywords:** mitral valve repair (MV repair), mitral regurgitation (MR), left atrial strain (LA strain), speckle tracking echocardiography, outcome

## Abstract

**Background:**

This study aimed to investigate the prognostic value of left atrial (LA) strain in patients with significant mitral regurgitation (MR) after surgical mitral valve (MV) repair.

**Methods:**

A total of 169 patients (age 55 ± 15 years, 88 men) with moderate or severe MR on echocardiogram at least 6 months after surgical MV repair for primary MR were studied. Two-dimensional, Doppler, and speckle tracking echocardiography including MR quantitative measures, chamber size, and LA strain were comprehensively analyzed. The primary outcome was a composite of cardiovascular death, heart failure hospitalization, and MV reoperation.

**Results:**

During a median of 44.4 months [interquartile range (IQR): 18.7–70.3 months] of follow-up, 44 patients (26%) experienced clinical events; these patients had greater MR volume, elevated mean diastolic pressure gradient and pulmonary artery systolic pressure, and enlarged chamber size compared with patients who did not experience events. Patients with events showed significantly lower LA strain [13.3% (IQR: 9.3–23.8%) vs. 24.0% (IQR: 13.1–31.4%), *p* = 0.003] and higher MR volume/LA strain [3.09 ml/% (IQR: 2.06–5.80 ml/%) vs. 1.57 ml/% (IQR: 1.04–2.72 ml/%), *p* < 0.001] than those without events. MR volume/LA strain was a good predictor of clinical outcomes (cut-off 1.57 ml/%, area under the curve 0.754, *p* < 0.001). On multivariable Cox proportional analysis, MR volume/LA strain was independently associated with clinical outcomes (hazard ratio: 1.269, 95% confidence interval: 1.109–1.452, *p* < 0.001) along with pulmonary artery systolic pressure.

**Conclusion:**

A measure of LA mechanical function relative to MR volume is associated with clinical outcomes in patients with significant MR after surgical MV repair.

## Introduction

Residual or recurrent mitral regurgitation (MR) after surgical mitral valve (MV) repair can occur even in excellent centers with a high MV repair success rate (1–3). Despite the relatively high recurrence of MR in patients who have undergone MV repair, treatment guidelines for these patients have not yet been established (4, 5). Therefore, when significant MR occurs after MV repair, clinicians usually decide the timing of reoperation depending on the severity of patient symptoms. In particular, significant MR after MV repair differs from primary native MR in terms of cardiac remodeling, so there is a limit to applying criteria such as increased left ventricular (LV) end-systolic dimension or decreased LV ejection fraction as indications for MV reoperation. Moreover, as the number of patients undergoing trans-catheter MV intervention for primary MR, including trans-catheter edge-to-edge repair (TEER), increases, there is a greater need for objective echocardiographic parameters to monitor the hemodynamic consequences of moderate to severe MR and determine the timing of the re-intervention or operation (6, 7).

Left atrial (LA) mechanical dysfunction assessed by two-dimensional speckle tracking echocardiography has been useful for predicting clinical outcomes in various cardiovascular diseases including atrial fibrillation, stroke, and heart failure (8–10). Theoretically, in patients who underwent MV repair, there is unavoidable restriction of the mitral annulus and little mitral stenosis physiology (11). Therefore, LA enlargement and mechanical dysfunction are more important than changes in LV in the presence of significant MR after MV repair. We hypothesized that LA strain, as a marker of LA mechanical function, can predict clinical outcomes in patients who present with significant MR after surgical MV repair. To prove this hypothesis, we sought to comprehensively analyze conventional and speckle tracking echocardiography and to identify predictors of clinical outcomes.

## Materials and methods

### Study design

**FIGURE 1:**
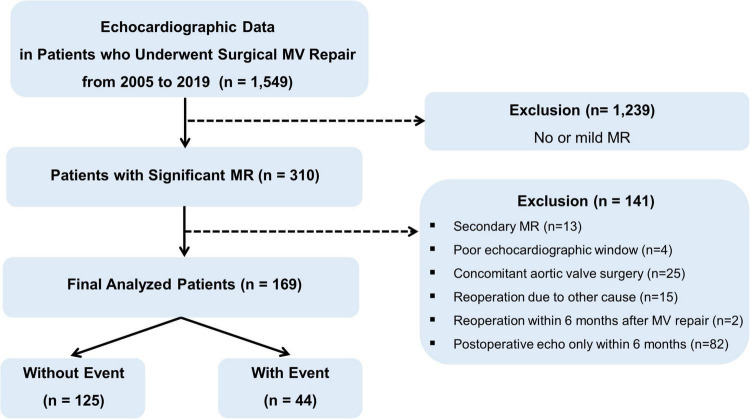
Study flow and design.

From the echocardiographic database from January 2005 to December 2019 at Severance Cardiovascular Hospital, Seoul, Korea, 1,549 patients who underwent surgical MV repair were identified. After exclusion of 1,239 patients with no or mild MR on echocardiogram, 310 patients with significant (at least moderate) MR, either residual or recurrent MR, were selected. Among them, a total of 169 patients with moderate or severe MR on echocardiogram at least 6 months after surgical MV repair for primary MR were enrolled, after excluding cases that met the following criteria: patients who underwent their MV repair for secondary MR; patients with poor-quality echocardiographic imaging; patients who underwent concomitant aortic valve surgery; patients who underwent reoperation for reasons other than MR; patients who underwent reoperation within 6 months of first MV repair; and patients without postoperative echocardiography 6 months after MV repair. The patients who underwent tricuspid annuloplasty and coronary artery bypass grafting surgery were included. The enrolled patients were divided into two groups according to the occurrence of clinical events (Figure 1). Clinical events were defined as a composite of cardiovascular deaths, heart failure hospitalization, and MV reoperation. All patients underwent postoperative TTE before discharge. Clinical and/or echocardiographic follow-up was performed 6 months after MV repair and annually thereafter in accordance with the institution’s follow-up protocol. Residual MR was defined when MR was confirmed in the postoperative TTE, and recurrent MR was defined as a case in which there was no MR in the postoperative TTE but confirmed in the follow-up TTE at least 6 months after surgery. The parameters of echocardiography used for the analysis are the parameters at the time of occurrence of significant MR in echocardiography at least 6 months after surgery. All laboratory or clinical information was collected from electronic medical records. A study protocol was developed according to the principles of the Declaration of Helsinki and was approved by the Institutional Review Board of Severance Hospital.

### Echocardiography

Transthoracic echocardiography was performed using a standard ultrasound machine (Vivid 7 or E9; GE Medical Systems; Wauwatosa, WI, Philips iE33 or Epiq7; Philips Healthcare; Netherlands) with a 2.5–3.5-MHz probe. Standard echocardiographic measurements were performed according to the recommendations of the American Society of Echocardiography guidelines (12). LA and LV volume, and LV ejection fraction (LVEF) were derived from standard apical 4- and 2-chamber views using the biplane method of disks. LA volume index was calculated by dividing LA volume by body surface area of patients (12). Total LA emptying fraction (TLAEF) was calculated as (maximal LA volume - minimal LA volume)/maximal LA volume × 100% (13). Maximal LA volume was defined as volume just before the MV closed and minimal LA volume was defined as volume just after the MV closed (13). Repaired MV function was assessed through a multi-parametric approach according to the guidelines of the American Society of Echocardiography using the following parameters (6, 14). Mean diastolic pressure gradient (MDPG) was assessed by continuous-wave Doppler. MR severity was determined based on volumetric methods including effective regurgitant orifice area (EROA), MR volume and MR fraction (6). Pulmonary artery systolic pressure (PASP) was determined using tricuspid regurgitation velocity and inferior vena cava diameter. In patients with atrial fibrillation, Doppler echocardiographic parameters were measured at the average of three cardiac cycles when the R-R interval was relatively regular.

### Two-dimensional speckle tracking echocardiography

Speckle tracking echocardiography was performed to evaluate LA and LV myocardial function. The analysis was performed offline using commercially available software (TomTec software; Image Arena 4.6, Munich, Germany). LA strain was obtained from apical 4- and 2-chamber images by semi-automatic endocardial border tracking and manual adjustment to optimize tracking (15). Among the 3 phases of atrial strain (reservoir, conduit, and contractile), LA strain was calculated by averaging the reservoir from 4- and 2-chamber images (15, 16). LV strain was obtained from acquired apical 4-chamber, apical 3-chamber, and apical 2-chamber views semi-automatically. LV global longitudinal strain (GLS) was calculated by averaging the peak strain value of 3 apical views (15, 17, 18). All data were performed and analyzed by two experienced individuals who were blinded to data analysis. To examine intra- and inter observer variability for LA and LV strain, two individuals repeated the analysis of the 20 consecutive patients. Since the amount of MR was diverse even in patients with significant MR, the ratio of MR volume to LA strain (MR volume/LA strain, ml/%) was used as a composite variable of MR severity and LA mechanical function.

### Statistical analysis

The study was followed from the index date, which a significant MR was diagnosed, until clinical events occurred or the study period ended on December 31, 2020. Continuous variables with a normal distribution were expressed as mean ± standard deviation and compared using Student’s *t*-test. Non-normally distributed variables were expressed as median [interquartile range (IQR)] and compared using Wilcoxon Rank Sum test. Categorical variables were presented as frequencies (percentages,%) and compared using the chi-square test or Fisher’s exact test. The predictive value of MR volume, LA strain, and MR volume/LA strain for the primary outcomes was calculated using receiver operating characteristic (ROC) analysis. Kaplan-Meier survival analyses and log-rank tests were used to compare clinical outcomes according to cutoff values for LA strain and MV volume/LA strain during the follow-up period. Univariate and multivariate Cox proportional analyses were used to obtain adjusted hazard ratios (HRs) with 95% confidence intervals (CIs) for evaluating the risk of events. The variables selected for entry into multivariate analysis were those with a *p*-value < 0.10 in univariate analysis. Multivariate analysis was conducted in two models. Model 1 was analyzed including LA strain, while model 2 was analyzed with MR volume/LA strain. A two-sided *p*-value of <0.05 was considered significant. Intra- and inter-observer variability values are expressed intra-class correlation coefficients (ICCs). Statistical analyses were conducted using SPSS software version 20 (SAS Institute Inc., Cary, NC, USA) and R software (version 3.6.3; R Foundation for Statistical Computing, Vienna, Austria).

## Results

### Clinical and echocardiographic characteristics

During a median 44.1 months [interquartile range (IQR): 18.7–70.3 months] of follow-up, among 169 patients, 44 patients (26%) experienced clinical events including 3 cardiovascular deaths, 10 heart failure hospitalizations, and 35 reoperations for MV (27 MV replacement and 8 redo-repair). Table 1 presents the baseline characteristics of the study population according to the occurrence of clinical events. Baseline characteristics did not differ between the two groups, except for higher prevalence of atrial fibrillation and lower estimated glomerular filtration rate in the event group. The most common etiology before MV repair was prolapse of MV. MR etiology before repair and device used for surgical MV repair were also comparable. However, LV and LA size were significantly larger in patients who experienced events compared to those who did not. There was no difference in LV function (LVEF and LV GLS) between the two groups, but LA function (LA strain and TLAEF) was significantly reduced in patients who experienced events compared to those who did not [LA strain: 13.3% (IQR: 9.3–23.8%) vs. 24.0% (IQR: 13.1–31.4%), *p* = 0.003]. MR was more severe in patients who experienced events, and TR was also more severe. Thus, MR volume/LA strain was remarkably higher in the event group than the non-event group [3.09 ml/% (IQR: 2.06–5.80 ml/%) vs. 1.57 ml/% (IQR: 1.04–2.72 ml/%), *p* < 0.001]. PASP was also significantly higher in patients who experienced events [37.5 mmHg (IQR: 28.0–46.3 mmHg) vs. 29.9 mmHg (IQR: 25.0–35.0 mmHg), *p* < 0.001, Table 2]. There are 75 patients who had residual significant MR. However, there were no significant difference for parameters for cardiac function assessed by TTE, except LV mass index between patients with residual MR and those with recurrent MR (Supplementary Table 1).

**TABLE 1:** Baseline characteristics of the study population.

	All (*n* = 169)	No clinical events (*n* = 125)	Experienced clinical events (*n* = 44)	*P-value*
Age, years	55.4 ± 14.6	54.9 ± 15.3	56.6 ± 12.5	0.511
Male sex, n (%)	88 (52.1)	63 (50.4)	25 (56.8)	0.577
Body mass index, kg/m^2^	22.7 ± 3.0	22.6 ± 2.9	23.1 ± 3.1	0.355
Hypertension, n (%)	62 (36.7)	42 (33.6)	20 (45.5)	0.222
Diabetes mellitus, n (%)	12 (7.1)	7 (5.6)	5 (11.4)	0.348
Chronic kidney disease, n (%)	20 (11.8)	11 (8.8)	9 (20.5)	0.074
Coronary artery disease, n (%)	28 (16.6)	18 (14.4)	10 (22.7)	0.297
Atrial fibrillation, n (%)	60 (35.5)	36 (28.8)	24 (54.5)	0.004
Charlson comorbidity index	1 (0-2)	1 (0-2)	1 (0-2)	0.762
Hemoglobin, g/dL	13.3 ± 2.7	13.5 ± 1.7	12.0 ± 2.6	0.003
Creatinine, mg/dL	1.0 ± 0.5	0.9 ± 0.5	1.1 ± 0.4	0.097
eGFR, ml/min/1.73 m^2^	83.1 ± 24.6	86.8 ± 23.4	73.3 ± 25.5	0.005
MR etiology before repair, n (%)				0.994
Rheumatic	12 (7.1)	9 (7.2)	3 (6.8)	
Prolapse	149 (88.2)	110 (88.0)	39 (88.6)	
Other	8 (4.7)	6 (4.8)	2 (4.5)	
Device used for MV repair, n (%)				0.887
C-ring	107 (63.3)	81 (64.8)	26 (59.1)	
D-ring	39 (23.1)	27 (21.6)	12 (27.3)	
Restrictive ring	23 (13.6)	17 (13.6)	6 (13.6)	
Residual significant MR	75 (44.4)	61 (48.8)	14 (31.8)	0.076

eGFR, estimated glomerular filtration rate; MR, mitral regurgitation; MV, mitral valve.

**TABLE 2:** Baseline echocardiographic characteristics of the study population.

	All (*n* = 169)	No clinical events (*n* = 125)	Experienced clinical events (*n* = 44)	*P-value*
LVEDD, mm	53.0 ± 6.1	52.0 ± 6.1	55.7 ± 5.1	<0.001
LVESD, mm	36.6 ± 6.4	36.0 ± 6.4	38.3 ± 6.4	0.043
LVEDV, ml	156.4 (122.2-191.8)	150.2 (118.6-179.8)	171.5 (133.6-207.4)	0.011
LVESV, ml	52.9 (41.1-71.1)	52.3 (40.0-68.6)	65.1 (44.4-84.1)	0.112
LVEF, %	61.7 ± 9.1	61.7 ± 8.6	61.6 ± 10.6	0.940
LV mass index, g/m^2^	101.3 (87.9-127.3)	97.8 (81.9-119.7)	110.1 (96.6-138.7)	0.017
LA volume index, ml/m^2^	57.0 (40.4-75.6)	52.5 (39.1-67.7)	75.9 (53.5-114.3)	<0.001
LVGLS, %	-15.8 ± 5.4	-15.7 ± 5.2	-16.2 ± 6.1	0.597
LA strain, %	19.9 (11.7-30.0)	24.0 (13.1-31.4)	13.3 (9.3-23.8)	0.003
TLAEF, %	35.0 (23.1-49.2)	40.6 (24.2-50.4)	26.7 (19.6-42.6)	0.013
MDPG, mmHg	4.4 (3.3-6.0)	4.0 (3.2-5.8)	5.0 (3.7-6.9)	0.031
MR severity				<0.001
Moderate, n (%)	129 (76.3)	107 (85.6)	22 (50.0)	
Severe, n (%)	40 (23.7)	18 (14.4)	22 (50.0)	
EROA, mm^2^	28.0 (23.0-34.5)	27.0 (23.0-32.3)	33.0 (27.0-45.8)	<0.001
MR volume, ml	36.7 (24.9-49.5)	34.1 (25.0-45.6)	51.7 (39.6-69.0)	<0.001
MR fraction, %	39.2 ± 12.2	36.5 ± 10.5	47.3 ± 13.7	<0.001
MR volume/LA strain, ml/%	1.98 (1.11-3.28)	1.57 (1.04-2.72)	3.09 (2.06-5.80)	<0.001
TR severity				<0.001
Mild, n (%)	54 (32.0)	34 (27.2)	20 (45.5)	
Moderate, n (%)	19 (11.2)	11 (8.8)	8 (18.2)	
Severe, n (%)	9 (5.4)	3 (2.4)	6 (13.7)	
PASP, mmHg	30.2 (26.0-39.1)	29.9 (25.0-35.0)	37.5 (28.0-46.3)	<0.001

LVEDD, left ventricular end-diastolic dimension; LVESD, left ventricular end-systolic dimension; LVEDV, left ventricular end-diastolic volume; LVESV, left ventricular end-systolic volume; LVEF, left ventricular ejection fraction; LV, left ventricular; LA, left atrial; GLS, global longitudinal strain; TLAEF, total left atrial ejection fraction; MDPG, mean diastolic pressure gradient; MR, mitral regurgitation; EROA, effective regurgitant orifice area; TR, tricuspid regurgitation; PASP, pulmonary artery systolic pressure.

### Left atrial mechanical function and clinical outcomes

**FIGURE 2:**
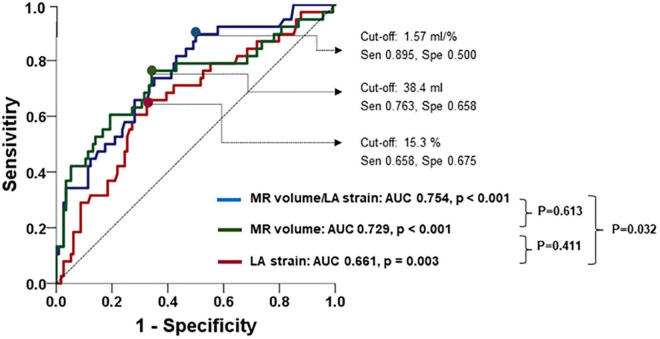
Predictive value of MR volume, LA strain, and their ratio for the occurrence of adverse clinical outcomes.

**FIGURE 3:**
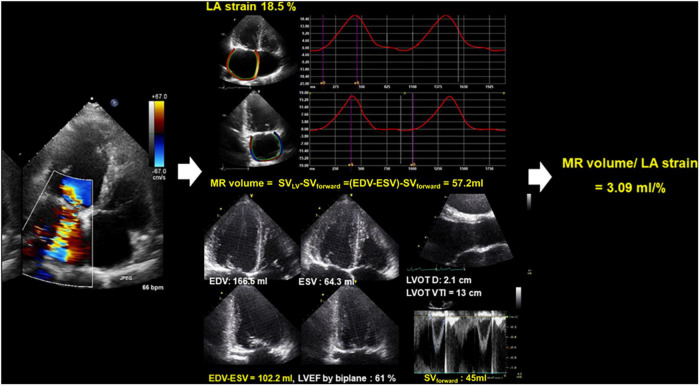
Representative case of LA mechanical dysfunction and the amount of MR in a patient with significant MR after surgical MV repair. EDV, end-diastolic volume; ESV, end-systolic volume; LA, left atrium; LVEF, left ventricular ejection fraction; LVOT D, left ventricular outflow tract distance; LVOT VTI, left ventricular outflow tract velocity time integral; MR, mitral regurgitation; SV, stroke volume.

Receiver operating characteristic was performed to evaluating the predictive value of LA mechanical function for clinical outcomes in this population. LA strain had good predictive performance for clinical outcomes [cut-off 15.3%, area under the curve (AUC) 0.661, *p* = 0.003]. The AUC for MR volume/LA strain was significantly larger than that of LA strain (cut-off 1.57 ml/%, AUC 0.754, *p* < 0.001) (comparison of both AUCs: *p* = 0.032) (Figure 2). Figure 3 is a representative case of reduced LA mechanical function and larger MR volume in a patient that experienced a clinical event.

**FIGURE 4:**
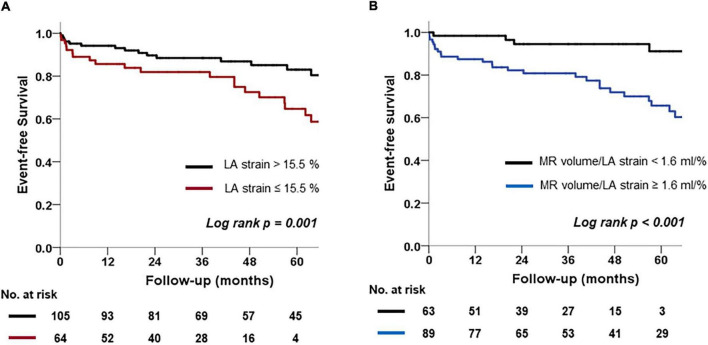
Kaplan-Meier analysis of freedom from clinical outcomes. **(A)** Comparison of two groups according to a cutoff value for LA strain. **(B)** Comparison of two groups according to a cutoff value for MR volume/LA strain.

Kaplan–Meier curves according to LA strain and MR volume/LA strain cutoffs are shown in Figure 4. Patients with reduced LA strain ≤15.5% had significantly more events compared with those with LA strain > 15.5% (Log-rank *p* = 0.001). In addition, patients with MR volume/LA strain > 1.6 ml/% exhibited poorer clinical outcomes than those with MR volume/LA strain ≤ 1.6 ml/% (Log-rank *p* < 0.001).

Table 3 displays univariate and multivariate Cox-proportional analysis results for clinical outcomes. MR severity, LV and LA size, LA strain, MR volume/LA strain, and PASP were significantly correlated with clinical outcomes in univariate analysis. After adjustment, LA strain was not an independent factor for clinical outcomes [hazard ratio (HR): 0.993, 95% confidence interval (CI): 0.945–1.043, *p* = 0.765], but MR volume/LA strain, a measure of LA mechanical dysfunction relative to MR, was independently associated with clinical outcomes (HR: 1.269, 95% CI: 1.109–1.452, *p* < 0.001), as was PASP. As a result of Cox-proportional analysis of clinical results by dividing residual MR and recurrent MR, MR volume/LA strain was a significant factor in both groups (Supplementary Table 2).

**TABLE 3:** Cox proportional analysis for clinical outcomes.

	Univariate			Model 1			Model 2		
	HR	95% CI	*P-value*	HR	95% CI	*P-value*	HR	95% CI	*P-value*
**Clinical characteristics**									
Charlson Comorbidity Index	1.186	0.944–1.49	0.144						
AF	2.362	1.300–4.290	0.005	1.156	0.450–2.974	0.763	0.859	0.375–1.967	0.719
**MV characteristics**									
EROA, cm^2^	1.049	1.029–1.070	<0.001						
MR volume, ml	1.037	1.023–1.052	<0.001	1.022	1.006–1.038	0.006			
MDPG, mmHg	1.099	0.978–1.235	0.114						
**Chamber characteristics**									
LVESV, ml	1.011	1.002–1.020	0.015	1.005	0.993–1.018	0.408			
LVEF, %	0.983	0.953–1.014	0.276						
LA volume index, ml/m^2^	1.013	1.007–1.018	<0.001	1.006	0.997–1.015	0.173	1.005	0.997–1.013	0.236
LV GLS, %	1.020	0.997–1.077	0.469						
LA strain, %	0.955	0.926–0.985	0.003	0.993	0.945–1.043	0.765			
**MR volume/LA strain, ml/%**	1.348	1.210–1.502	<0.001				1.269	1.109–1.452	<0.001
**PASP, mmHg**	1.065	1.042–1.088	<0.001	1.034	1.005–1.063	0.020	1.040	1.014–1.066	0.002

AF, atrial fibrillation; EROA, effective regurgitant orifice area; MR, mitral regurgitation; MDPG, mean diastolic pressure gradient; LVESV, left ventricular end-systolic volume; LVEF, left ventricular ejection fraction; LA, left atrial; LV GLS, left ventricular global longitudinal strain; PASP, pulmonary artery systolic pressure.

### Intra-observer and inter-observer variability

To assess variability for LA strain and LV GLS, two individuals repeated the analysis of the 20 consecutive patients. The ICCs revealed excellent reliability for LA strain and LV GLS, with the ICCs generally > 0.9. The intra- and inter-observer ICCs for LA strain were 0.97 (0.93–0.98) and 0.91 (0.79–0.96) and those for LV GLS were 0.99 (0.97–1.00) and 0.94 (0.88–0.98), respectively.

## Discussion

The main findings in this study are as follows: (1) in patients with significant MR after surgical MV repair, patients with clinical events exhibited decreased LA strain compared to those without clinical events; (2) LA strain assessed by two-dimensional speckle tracking echocardiography was a good predictor of clinical outcomes; (3) an index of reduced LA mechanical function relative to the amount of MR was independently associated with clinical outcomes after controlling for confounding factors. These findings suggest that an LA strain-based echocardiographic parameter might be useful for risk stratification and decision making for re-intervention in patients with significant MR after MV repair.

In the early phase of MR, LA and LV compensate for volume overload, but if chronic volume overload continues, further LA and LV remodeling occurs, resulting in cardiac chamber dilatation and dysfunction (19, 20). After successful MV surgery, reverse remodeling occurs at least 6 months (21, 22). While some patients recover from LA and LV mechanical dysfunction, other patients exhibit irreversible LA and LV damage accompanied by cardiac fibrosis (21, 23). LA and LV enlargement are commonly observed in patients who undergo MV repair. Moreover, absolute value of LA strain and LV GLS assessed by speckle tracking echocardiography in patients treated for MV are usually lower than absolute reference values (16, 24, 25). Therefore, it is difficult to decide the optimal timing of re-intervention or repeat surgery using the same chamber enlargement or dysfunction criteria used for *de novo* MR patients. Many studies have suggested that the presence of LA and LV mechanical dysfunction before MV surgery have prognostic implications for MV surgery in patients with chronic severe MR (26–28). However, there are no large-scale studies on which factors, including LA and LV mechanical dysfunction, have prognostic implications when significant MR occurs in patients undergoing MV repair.

The severity of valvular disease and changes in chamber function that are directly affected by severity are very important factors in determining outcomes (29). Therefore, rather than independently considering the severity of valvular disease or changes in chamber function, we focused on the fact that a robust correlation with outcome can be expected if indexed with a numerical value that reflects these two together. For example, when a large amount of water is put in a balloon, if the balloon has good distensibility, the balloon will not burst or overflow with water. Therefore, the correlation with outcomes was identified through RV/LAGLS, a new parameter representing the change in MR volume, a quantitative assessment of MR severity and LV function. As a result, in this study, it was confirmed that small RV/low LAGLS and large RV/high LAGLS showed similar outcome rates (Supplementary Figure 1B). After all, even in the similar MR severity, if the LA function is good, there may be fewer events, and conversely, if the LA function is low, many events may occur. This study showed that RV/LAGLS, as parameter that reflects LA function in MR severity, will play an important role in predicting prognosis.

Previous studies have reported the incidence and outcomes of recurrence of MR after surgical MV repair (1, 2). A study in patients with degenerative MV disease demonstrated significant recurrence of MR (3.7% annually) during 7 years of follow-up after MV repair (1). In another long-term study of 1,234 patients who underwent MV repair, 60.4% of patients did not experience any events for approximately 20 years; 12.5% had moderate or severe MR, and only 4.6% underwent re-operation for MR (2). Recently, as various trans-catheter interventions as well as surgical MV repair have been performed as alternative treatments for severe MR, there is growing interest in significant MR after MV repair (30–33). Chronic significant MR after MV repair differs from primary native MR in terms of cardiac remodeling, so there is a limit to applying criteria such as increased LV dimensions and LV dysfunction when making decisions regarding MV reoperation.

The present study suggests important points for clinical decision-making. First, increased PASP in patients with significant MR after MV repair is an important prognostic factor, as proven in patients with chronic native MR (34). Second, even in patients with significant MR, quantitative measurement of MR amount is helpful for risk stratification. Third, an index of LA mechanical dysfunction relative to MR amount is an important prognostic factor in patients with significant MR after MV repair. These findings will be clinically useful for decision-making and clinical follow-up for significant MR after MV repair.

### Limitations

Our study has some limitations. First, as this was a single-center, retrospective study, there are fundamental limitations. Further multicenter studies and large-scale cohorts are necessary. Second, the postoperative echocardiography period at which significant MR was observed varied, and both residual MR and recurrent MR were included in this study. However, essentially, both residual and recurrent MR are MV regurgitation, and if there are factors affecting the clinical outcome at the time when significant MR is confirmed, appropriate intervention is required regardless of residual or recurrent. Routine institutional echocardiographic follow-up after surgical MV repair was conducted 6 months after the surgery and annually thereafter. Therefore, we believe that these results meet the research objective of identifying clinically meaningful echocardiographic parameters in the presence of significant MR in patients with repaired MV. Third, LA mechanical dysfunction was an important test variable, but LA strain did not show an independent association with clinical outcomes in multivariate analysis. However, MR volume/LA strain, which is an index of MR amount in LA mechanical dysfunction, was independently associated with clinical outcome. Since our study targeted moderate or more MR, we think that it is acceptable result that the measurement value for the degree of MR is important together with the LA mechanical function.

## Conclusion

Left atrial mechanical dysfunction relative to increased MR amount is associated with poor clinical outcomes in patients with significant MR after surgical MV repair.

## Data availability statement

The original contributions presented in this study are included in the article/Supplementary material, further inquiries can be directed to the corresponding author.

## Ethics statement

This study was reviewed and approved by the Institutional Review Board of Severance Hospital. The study protocol was developed according to the principles of the Declaration of Helsinki. Written informed consent for participation was not required for this study in accordance with the national legislation and the institutional requirements.

## Author contributions

S-EK and CS: conception, planning and/or conducting the study, and drafting the manuscript. S-EK, D-YK, JS, IC, G-RH, J-WH, and CS: collecting and interpreting data. CS: guarantor of the article. All authors contributed to the article and approved the submitted version.

## Supplementary material

Supplementary Figure 1 Kaplan–Meier analysis of freedom from clinical outcomes. **(A)** Comparison according to MR severity. **(B)** Comparison of four groups according to RV and LAGLS.

## Abbreviations

CI: confidence interval
EROA: effective regurgitant orifice area
GLS: global longitudinal strain
HR: hazard ratio
ICC: intra-class correlation coefficients
LA: left atrial
LV: left ventricle
LVEF: left ventricular ejection fraction
MDPG: mean diastolic pressure gradient
MR: mitral regurgitation
MV: mitral valve
PASP: pulmonary artery systolic pressure
ROC: receiver operating characteristic
TEER: transcatheter edge-to-edge repair
TLAEF: total LA emptying fraction.
