# Supplementary material for: Left atrial strain and clinical outcome in patients with significant mitral regurgitation after surgical mitral valve repair
Source: Front Cardiovasc Med. 2022 Oct 4;9:985122. doi: 10.3389/fcvm.2022.985122 (PMC9577607; doi:10.3389/fcvm.2022.985122)
Supplement: Supplementary file 1 [file Data_Sheet_1.docx]

**Supplementary Table 1. Cardiac function assessed by echocardiography between residual MR and recurrent MR populations**

|  | **Residual MR**  **(n =75)** | **Recurrent MR**  **(n = 94)** | **p-value** |
| --- | --- | --- | --- |
| LVEDD, mm | 53.5 ± 6.8 | 52.6 ± 5.5 | 0.325 |
| LVESD, mm | 37.1 ± 7.4 | 36.2 ± 5.6 | 0.388 |
| LVEDV, ml | 156.7 (119.1-189.9) | 155.5 (127.6-190.7) | 0.801 |
| LVESV, ml | 54.2 (40.2-70.3) | 52.3 (41.9-70.8) | 0.857 |
| LVEF, % | 60.7 ± 9.9 | 62.4 ± 8.4 | 0.221 |
| LV mass index, g/m^2^ | 110.7 (89.3-132.2) | 98.8 (82.3-117.9) | 0.030 |
| LA volume index, ml/m^2^ | 54.4 (40.0-70.2) | 59.4 (41.0-80.30 | 0.475 |
| LV GLS, % | -15.2 ± 5.7 | -16.3 ± 5.2 | 0.175 |
| LA strain, % | 19.9 (13.1-29.2) | 21.6 (11.6-31.3) | 0.794 |
| TLAEF, % | 37.4 (26.0-47.3) | 33.2 (19.9-50.1) | 0.331 |
| MDPG, mmHg | 4.5 (3.3-6.2) | 4.0 (3.3-5.9) | 0.617 |
| MR severity |  |  | 0.927 |
| Moderate, n (%) | 58 (77.3) | 71 (75.5) |  |
| Severe, n (%) | 17 (22.7) | 23 (24.5) |  |
| EROA, mm^2^ | 30 (24-36) | 27 (22-34) | 0.248 |
| MR volume, ml | 35.3 (23.8-47.1) | 37.9 (28.5-51.2) | 0.330 |
| MR fraction, % | 36.9 (31.7-46.3) | 38.1 (32.6-46.5) | 0.413 |
| MR volume/LA strain, ml/% | 1.99 (1.11-2.85) | 1.97 (1.15-3.58) | 0.501 |
| TR severity |  |  | 0.105 |
| Mild, n (%) | 26 (34.7) | 28 (29.8) |  |
| Moderate, n (%) | 9 (12.0) | 10 (10.6) |  |
| Severe, n (%) | 0 (0.0) | 9 (9.6) |  |
| PASP, mmHg | 30 (26-37) | 30.6 (26-40) | 0.539 |

LVEDD, left ventricular end-diastolic dimension; LVESD, left ventricular end-systolic dimension; LVEDV, left ventricular end-diastolic volume; LVESV, left ventricular end-systolic volume; LVEF, left ventricular ejection fraction; LV, left ventricular; LA, left atrial; GLS, global longitudinal strain; TLAEF, total left atrial ejection fraction; MDPG, mean diastolic pressure gradient; MR, mitral regurgitation; EROA, effective regurgitant orifice area;; TR, tricuspid regurgitation; PASP, pulmonary artery systolic pressure

**Supplementary Table 2. Cox proportional analysis according to type of MR.**

|  | **Recurrent MR (n=94)** | | **Residual MR (n=75)** | |
| --- | --- | --- | --- | --- |
|  | **HR (95% CI)** | **p-value** | **HR (95% CI)** | **p-value** |
| **Reg. V., /LA strain, ml/%** | 1.321 (1.161-1.503) | <0.001 | 1.328 (1.093-1.615) | 0.004 |
| **PASP, mmHg** | 1.072 (1.047-1.098) | <0.001 | 1.022 (0.974-1.072) | 0.378 |
| **LA strain, %** | 0.953 (0.919-0.988) | 0.008 | 0.967 (0.914-1.023) | 0.242 |

Reg. V., regurgitant volume; PASP, pulmonary artery systolic pressure
