# Supplementary figures and images for: Left atrial strain and clinical outcome in patients with significant mitral regurgitation after surgical mitral valve repair
Source: Front Cardiovasc Med. 2022 Oct 4;9:985122. doi: 10.3389/fcvm.2022.985122 (PMC9577607; doi:10.3389/fcvm.2022.985122)

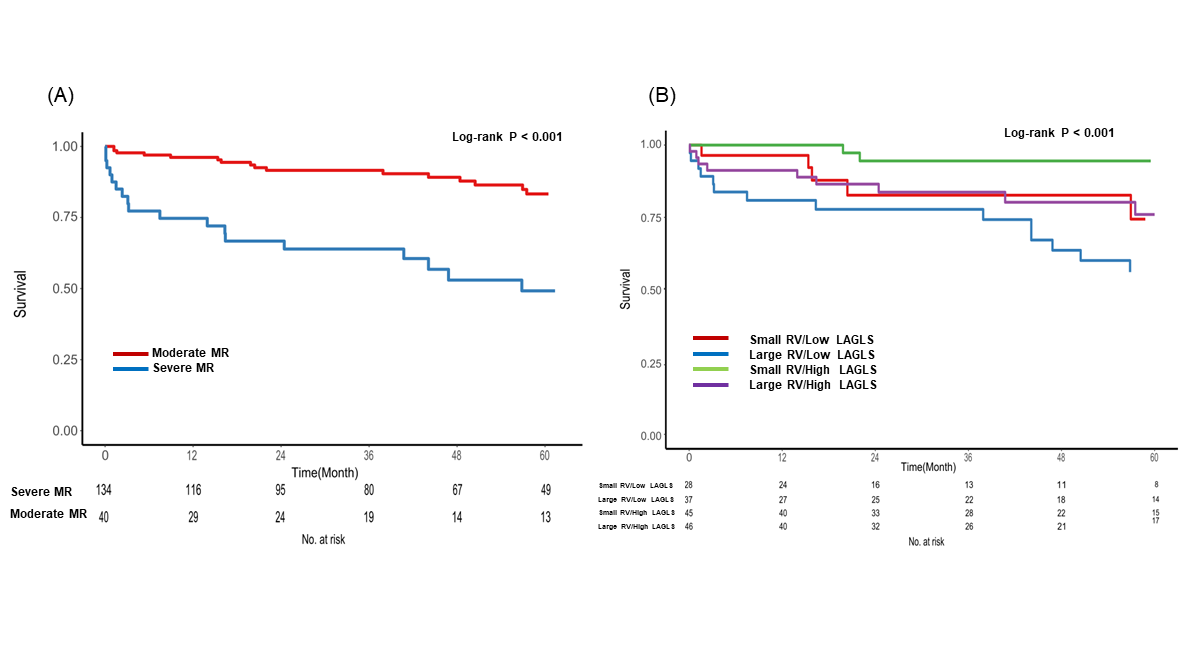

Supplement: Supplementary Figure 1 — Kaplan–Meier analysis of freedom from clinical outcomes. (A) Comparison according to MR severity. (B) Comparison of four groups according to RV and LAGLS. [file Image_1.TIF]
